# MiR-193a-3p and miR-193a-5p suppress the metastasis of human osteosarcoma cells by down-regulating Rab27B and SRR, respectively

**DOI:** 10.1007/s10585-016-9783-0

**Published:** 2016-02-25

**Authors:** Youguang Pu, Fangfang Zhao, Wenjing Cai, Xianghui Meng, Yinpeng Li, Shanbao Cai

**Affiliations:** Cancer Epigenetics Program, Anhui Cancer Hospital, Hefei, 230031 Anhui China; Indiana University School of Medicine, Indianapolis, IN 46202 USA; Department of Orthopedic Surgery, Anhui Cancer Hospital, Hefei, 230031 Anhui China; Xinxiang Medical University, Xinxiang, 453000 Henan China

**Keywords:** Rab27B, SRR, MiR-193a-3p and miR-193a-5p, Osteosarcoma, Metastasis

## Abstract

MicroRNAs have been identified as key players in the development and progression of osteosarcoma, which is the most common primary malignancy of bone. Sequencing-based miR-omic and quantitative real-time PCR analyses suggested that the expression of miR-193a-3p and miR-193a-5p was decreased by DNA methylation at their promoter region in a highly metastatic osteosarcoma cell line (MG63.2) relative to their expression in the less metastatic MG63 cell line. Further wound-healing and invasion assays demonstrated that both miR-193a-3p and miR-193a-5p suppressed osteosarcoma cell migration and invasion. Moreover, introducing miR-193a-3p and miR-193a-5p mimics into MG63.2 cells or antagomiRs into MG63 cells confirmed their critical roles in osteosarcoma metastasis. Additionally, bioinformatics prediction along with biochemical assay results clearly suggested that the secretory small GTPase Rab27B and serine racemase (SRR) were direct targets of miR-193a-3p and miR-193a-5p, respectively. These two targets are indeed involved in the miR-193a-3p- and miR-193a-5p-induced suppression of osteosarcoma cell migration and invasion. MiR-193a-3p and miR-193a-5p play important roles in osteosarcoma metastasis through down-regulation of the Rab27B and SRR genes and therefore may serve as useful biomarkers for the diagnosis of osteosarcoma and as potential candidates for the treatment of metastatic osteosarcoma.

## Introduction

Osteosarcoma is the most common primary bone malignancy in children and young adults [1, 2]. Approximately 80 % of osteosarcoma patients have metastatic disease at the time of diagnosis, and metastasis is a consistent problem in tumor prognosis and treatment [3]. The molecular mechanisms that lead to the development and metastasis of osteosarcoma are poorly understood [4–6]. Further exploration of this area will help in the development of effective strategies in the diagnosis, treatment and prognosis of osteosarcoma. Previous studies have established a highly tumorigenic and metastatic human osteosarcoma cell line, MG63.2, derived from the less metastatic parental MG63 cell line [7]. The MG63.2 cell line facilitates the exploration of crucial players in osteosarcoma metastasis [8].

MicroRNAs (miRNAs), a class of small non-coding RNA molecules, play critical roles in a variety of biological events, including development, cell proliferation and cell differentiation [9–11]. MiRNAs negatively regulate gene expression by binding to the 3′-untranslated regions (UTRs) of the corresponding target mRNAs of protein-coding genes, thereby leading to mRNA degradation or translation inhibition [12–15]. Multiple miRNAs are involved in the invasion and metastasis of different types of cancers, including gastric cancer, breast cancer, hepatocellular carcinoma and colorectalcancer [16–19]. In osteosarcoma, a few miRNAs have been reported to regulate metastasis. For instance, miR-16 inhibits cell proliferation by targeting IGF1R and the Raf1-MEK1/2-ERK1/2 pathway in osteosarcoma [20], and miR-802 promotes osteosarcoma cell proliferation by down-regulating the p27 cell-cycle inhibitor [21]. Moreover, miR-212 inhibits osteosarcoma cell proliferation and invasion by down-regulating Sox4 [22]. However, the prospects of these miRNAs in the clinical treatment of osteosarcoma have not been evaluated. Novel miRNAs that may potentially serve as new therapeutic targets or biomarkers must urgently be identified.

In the present study, we aimed to identify novel miRNAs associated with osteosarcoma metastasis. From a sequencing-based miR-omic study and referred to the relevant literatures, we found two miRNAs, miR-193a-3p and miR-193a-5p, that play a role in osteosarcoma metastasis. These two miRNAs, which are derived from the same precursor, have different sequences and therefore target different mRNAs [23]. These miRNAs have been implicated in the tumorigenesis and progression of several types of cancer [24–28]. In addition, we identified the target genes of miR-193a-3p and miR-193a-5p, and we proposed the potential pathway that might be associated with osteosarcoma metastasis. Therefore, miR-193a-3p and miR-193a-5p, as well as their targets, might serve as useful biomarkers for the diagnosis of osteosarcoma and as potential candidates for the treatment of metastatic osteosarcoma.

## Materials and methods

### Cell lines and culture

The MG63 osteosarcoma cell line was purchased from the American Type Culture Collection (ATCC; NO. CRL-1427), and the MG63.2 cell line was kindly provided by Dr. Luu from the University of Chicago [7]. The cells were cultured in Dulbecco’s modified Eagle’s medium (DMEM; Invitrogen, Carlsbad, CA, USA) supplemented with 10 % fetal bovine serum (FBS; Invitrogen) and 1 % glutamine at 37 °C in 5 % CO_2_.

### RNA-seq analysis

RNA-seq analysis was performed by BGI-Tech of China, and RNA-seq library preparation and sequencing were performed by BGI (Shenzhen, China). Following purification, RNA was fragmented using divalent cations at an elevated temperature, and first-strand cDNA was synthesized using random hexamer primers and Superscript TMIII (Invitrogen™, Carlsbad, CA, USA). Second-strand cDNA was synthesized using buffer, dNTPs, RNaseH, and DNA polymerase I. Short fragments were purified with a QiaQuick PCR extraction kit (Qiagen) and resolved with EB buffer for end reparation and poly (A) addition. The short fragments were subsequently connected using sequencing adapters. After agarose gel electrophoresis, suitable fragments were used as templates for PCR amplification. During the QC steps, an Agilent 2100 Bioanaylzer and an ABI StepOnePlus Real-Time PCR System were used in quantification and qualification of the sample library. Finally, the library (200 bp insert) was sequenced using Illumina HiSeq 2000 (Illumina Inc., San Diego, CA, USA). The single-end library was prepared following the protocol of the IlluminaTruSeq RNA Sample Preparation Kit (Illumina).

### Transfection of mimics, antagomiRs, siRNAs and overexpression plasmids

The mimics, antagomiRs, siRNAs, scramble sequence negative control (NC) and riboFECT CP transfection kits were supplied by Guangzhou Ribobio (Guangzhou, China). The mammalian expression constructs for GFP-tagged Rab27B (EX-Q0376-M98-5) and serine racemase (SRR) (EX-W0524-M98-5) were supplied by Guangzhou Fulengen (Guangzhou, China). Transfection of the ribonucleic acid reagents or plasmids mentioned in this paper as well as the reporter plasmids in the Cignal Finder™ Pathway Reporter Arrays (SABiosciences, USA) was performed according to the manufacturer’s instructions. The following sequences were used in this study:

- si-Rab27B
- 5′-CCCUGAUACUGUCAAUGGUdTdT-3′;
- 3′-dTdTGGGACUAUGACAGUUACCA-5′;
- si-SRR
- 5′-UGCCGUCAGAAGCUUGGUUdTdT-3′;
- 3′-dTdTACGGCAGUCUUCGAACCAA-5′;
- hsa-miR-193a-5p
- antagomiR: 5′-UCAUCUCGCCCGCAAAGACCCA-3′;
- mimics:
- sense, 5′-UGGGUCUUUGCGGGCGAGAUGA-3′
- antisense, 5′-UCAUCUCGCCCGCAAAGACCCA-3′;
- hsa-miR-193a-3p
- antagomiR: 5′-ACUGGGACUUUGUAGGCCAGUU-3′;
- mimics:
- sense, 5′-AACUGGCCUACAAAGUCCCAGU-3′
- antisense, 5′-ACUGGGACUUUGUAGGCCAGUU-3′.

Mimics were composed of double-stranded RNA with no chemical modifications. The 3′ ends of the antagomiR and agomiR oligo nucleotides were conjugated to cholesterol, and all the bases were 2′-O methylated (OMe). All of the oligo nucleotides were deprotected, desalted and purified by high-performance liquid chromatography.

### Luciferase reporter assay

The full-length 3′-UTR of human SRR (1386 bp) and a partial 3′-UTR of Rab27B (1034 bp; 830–1863 from a 6103 bp full-length sequence) containing miR-193a-5p- and miR-193a-3p-binding sites, respectively, were cloned downstream of the firefly luciferase gene in pGL3 (Invitrogen) to construct pGL3-luc-Rab27B and pGL3-luc-SRR, respectively. All of the constructs were confirmed by restriction digestion. Cells were seeded into 96-well plates at approximately 1 × 10^4^ cells per well and transfected with a mixture of 50 ng of pGL3-luc-Rab27B or pGL3-luc-SRR, 5 ng of Renilla luciferase and 5 pmol mimics or NC nucleotides using the riboFECT CP transfection reagents according to the manufacturer’s instructions. Both firefly and Renilla luciferase activities were measured 24 h after transfection using a Dual-Luciferase Reporter Assay System (Promega) on the Promega GloMax 20/20 luminometer. The relative firefly luciferase activities were normalized with the Renilla luciferase activities as a control for transfection efficiency.

### Pathway luciferase reporter constructs

The NC construct, which encodes firefly luciferase under the control of a basal promoter element (TATA box) without any additional transcriptional response elements, is critical for identifying specific effects and determining background reporter activity. The reporter construct encodes the firefly luciferase reporter gene under the control of a basal promoter element (TATA box) joined to tandem repeats of a specific transcriptional response element. The positive control construct is a mix of constructs that constitutively express GFP, firefly luciferase and Renilla luciferase. The analysis was performed according to the manufacturer’s instruction (SABiosciences). Briefly, the cells were triple transfected with each firefly luciferase reporter construct in combination with the Renilla luciferase construct using the riboFECT CP transfection reagent, and both luciferase activities in cell extracts at 24 h after transfection were measured using the Promega Dual-Luciferase Reporter assay on the PromegaGloMax 20/20 luminometer. Firefly luciferase activities from each set were normalized to the Renilla luciferase activity to control for inter-transfection bias. The relative luciferase activities (luciferase unit) of the pathway reporter over the negative control in the transfected cells were calculated as a measure of the pathway activity.

### RNA analysis

Total RNA was isolated using the TRIzol reagent (Tiangen Biotech Co., Ltd., Beijing, China). For mRNA analysis, cDNA was synthesized from total RNA by oligo-dT priming by using a primeScript RT reagent kit (Tiangen Biotech Co., Ltd.). The Rab27B and SRR mRNA levels were measured by quantitative real-time polymerase chain reaction (qRT-PCR) using gene-specific fluorescent TaqMan probes together with β-actin using a different fluorescent probe (provided by ShingGene, Shanghai, China) on an FTC-3000P PCR instrument (FUNGLYN BIOTECH INC, Toronto, ON, Canada). For miRNA analysis, cDNA was synthesized with specific stem-loop primers and quantified by using a SYBR Green-based RT-PCR assay, and normalization to the U6 reads for miRNA or to β-actin for mRNA was performed using the $2^{{ - \Updelta \Updelta {\text{C}}_{\text{t}} }}$ method before calculating the relative mRNA levels between MG63 and MG63.2 cells. The following primers and probes for complementary DNA synthesis and qRT-PCR analysis were used:

- hRab27BF, 5′-GGGACACTGCGGGACAAG-3′;
- hRab27BR, 5′-CAGTTGGCTCATCCAGTTTCTG-3′;
- hRab27B probe, 5′-CY5-CGGTTCCGGAGTCTCACCACTGC-3′;
- hSRRF, 5′-TTTAGAAAGGAAGCCGAAAGC-3′;
- hSRRR, 5′-GTCTGGGGCACCACAATATAAG-3′;
- hSRR probe, 5′-ROX-CTCACAGCAGTGGAAACCATGGCC-3′;
- hACTBF, 5′-GCCCATCTACGAGGGGTATG-3′;
- hACTBR, 5′-GAGGTAGTCAGTCAGGTCCCG-3′;
- hACTB probe, 5′-HEX-CCCCCATGCCATCCTGCGTC-3′.

### Western blotting analysis

Cell lysates in 1× SDS loading buffer (60 mM Tris–HCl, pH6.8; 2 % SDS; 20 % glycerol; 0.25 % bromophenol blue; and 1.25 %2-mercaptoethanol) were incubated at 100 °C for 10 min to facilitate sample loading for conventional western blotting analysis. Anti-Rab27B (13412-1-AP), anti-SRR (17955-1-AP) and anti-GAPDH (60004-1-lg) antibodies were provided by San Ying Biotechnology, China. The target proteins were then probed with an anti-rabbit IgG peroxidase-conjugated antibody (LP1001b) (San Ying Biotechnology, China) and detected by an enhanced chemiluminescence reaction (ThermoFisher Scientific, Waltham, MA, USA). The relative protein levels were quantified using densitometry with a Gel-Pro Analyzer (Media Cybernetics, Rockville, MD, USA). The intensities of the target bands relative to GAPDH were quantified by densitometry, and these values are indicated under each band.

### Bisulfite sequencing PCR (BSP) analysis

Genomic DNA was isolated using a standard phenol/chloroform purification method, qualified via agarose gel electrophoresis, and treated with an ammonium bisulfate-based bisulfite conversion method [29, 30]. The PCR fragments from the converted DNA were sequenced and analyzed. Raw sequence data files were processed, and the area ratio (%) of C to C+T of the primary CpG dinucleotide was calculated as the percentage of methylation and was then plotted.

### Wound-healing assays

For cell motility assays, cells stably expressing mimics, antagomiRs or NC were seeded in 24-well plates and cultured to near confluence. After 6 h of culture in DMEM without FBS, a linear wound was carefully made using a sterile 10 µl pipette tip across the confluent cell monolayer, and the cell debris was removed by washing with phosphate-buffered saline. The cells were incubated in DMEM plus 10 % FBS, and the wounded monolayers were then photographed at 0, 8, 24 and 48 h after wounding.

### In vitro invasion assays

Cell invasion assays were performed in a 24-well plate with 8 mm pore size chamber inserts (Corning, New York, NY, USA). For invasion assays, 1 × 10^4^ cells stably expressing mimics, antagomiRs or NC were placed into the upper chamber in each well with the matrigel-coated membrane, which was diluted in serum-free culture medium. In the assay, cells were suspended in 100 µl of DMEM without FBS when they were seeded into the upper chamber. In the lower chamber, 500 µl of DMEM supplemented with 10 % FBS was added. After incubation for 30 h at 37 °C and 5 % CO_2_, the membrane inserts were removed from the plate, and non-invading cells were removed from the upper surface of the membrane. Cells that moved to the bottom surface of the chamber were stained with 0.1 % crystal violet for 30 min. The cells were then imaged and counted in at least five random fields using a CKX41 inverted microscope (Olympus, Tokyo, Japan). The assays were conducted three independent times.

### Bioinformatics analysis

RAB27B, SRR and key pathway genes (TGFβ: TGFB1; MYC/Max: MYC; and ATF2/ATF3/ATF4: ATF2, ATF3, and ATF4) were used as query genes to predict potential interactions in the GeneMANIA database (http://genemania.org/). GeneMANIA uses a fast heuristic algorithm to integrate functional association networks containing all input genes. To simplify the model, the shortest paths between query genes were extracted from the networks. Those interactions reported in the literature were chosen first to obtain the true functional role of each interaction in the connected network of all query genes.

### Statistical analysis

Data are presented as the means, and error bars indicate the standard deviation (SD). All statistical analyses were performed with Excel (Microsoft, Redmond, WA, USA). Two-tailed Student’s *t* test, one-way analysis of variance (ANOVA) or the Mann–Whitney *U* test was used to calculate statistical significance. A *P* value of <0.05 was considered to be significant.

## Results

### MiR-193a-3p and miR-193a-5p are hypermethylated and down-regulated in the highly metastatic MG63.2 osteosarcoma cell line

**Fig. 1:**
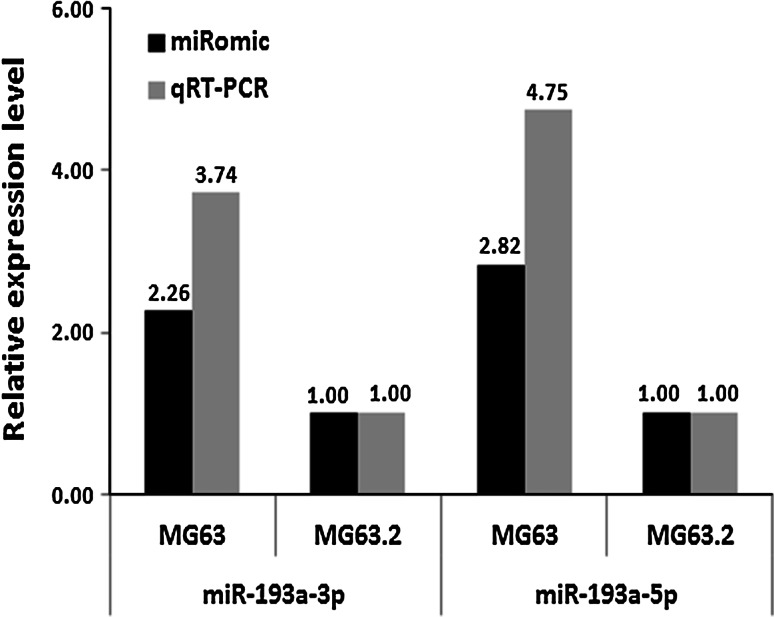
The relative miR-193a-3p and miR-193a-5p expression levels (fold change) in the MG63 and MG63.2 cell lines as measured by miR-omic and qRT-PCR analyses are shown in the *plot*

**Fig. 2:**
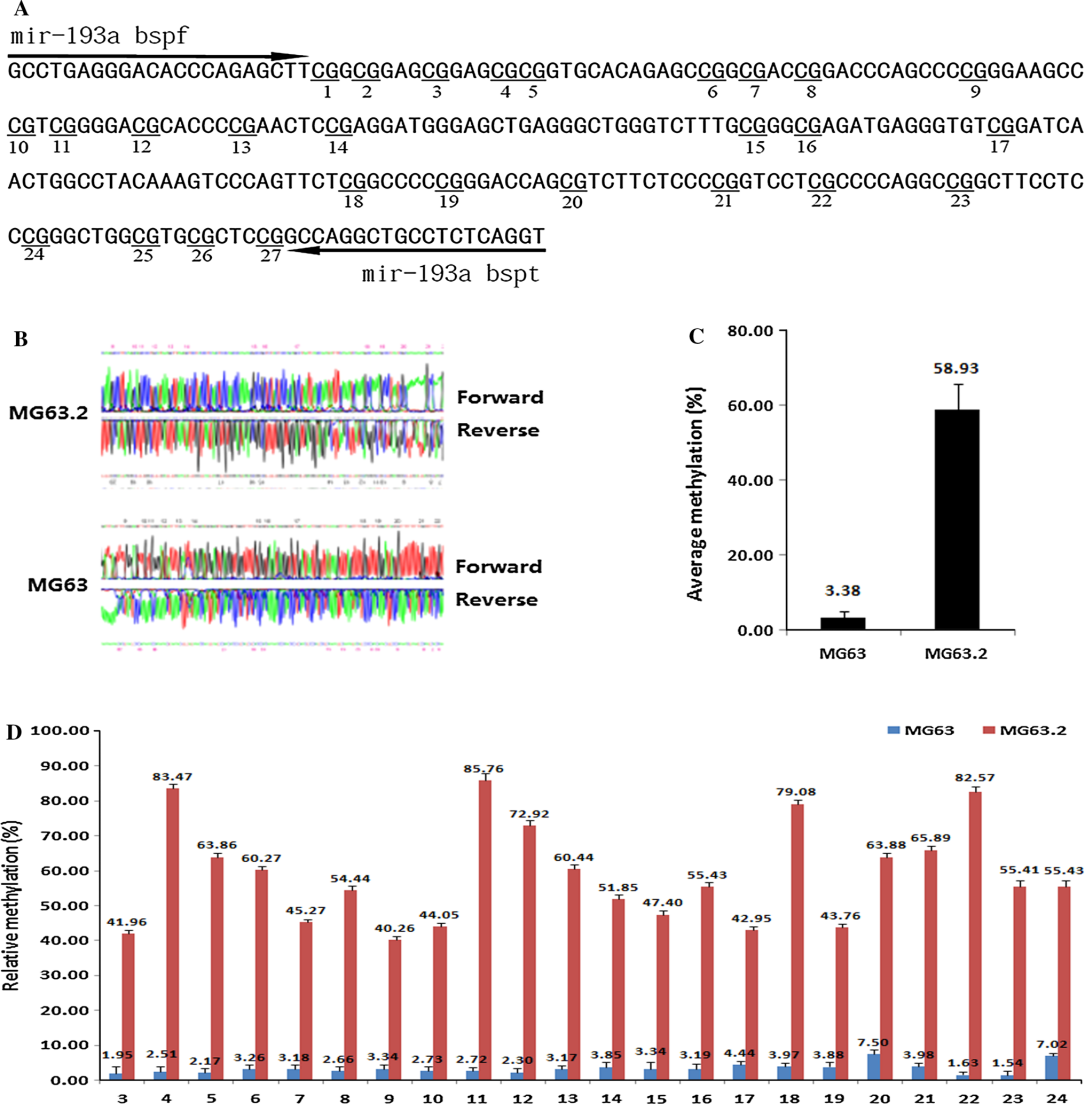
Differential methylation of the miR-193a gene in MG63 versus MG63.2 cells. The CpG dinucleotides and the BSP primers of the miR-193a gene are shown (**a**). The original sequencing results of the bisulfite-converted DNA are shown (**b**). The percentage of CpG methylation is summarized in the plot (**c**, **d**)

MiR-193a-3p and miR-193a-5p have been characterized as tumor suppressors in several types of cancer such as non-small cell lung cancer (NSCLC) [24, 28], prostate cancer [21], breast cancer [31], head and neck squamous cell carcinomas [32], and colorectal cancer [33]. However, the roles of miR-193a-3p and miR-193a-5p in osteosarcoma cells remain unclear. According to a sequencing-based miR-omic study in two human osteosarcoma cell lines (weakly metastatic MG63 cells and highly metastatic MG63.2 cells), both miR-193a-3p and miR-193a-5p were found to be among the top differentially expressed miRs. Quantitative real-time polymerase chain reaction (qRT-PCR) analyses verified that miR-193a-3p and miR-193a-5p expression was lower in the highly metastatic MG63.2 cells than in the weakly metastatic MG63 cells (the expression ratios of miR-193a-3p and miR-193a-5p in MG63.2 with MG63 were 1.00:3.74 and 1.00:4.75, respectively; Fig. 1). To further investigate the underlying mechanism of decreased miR-193a-3p and miR-193a-5p expression in highly metastatic MG63.2 cells, the methylation status of the miR-193a promoter regions in both MG63 and MG63.2 cells were assessed using BSP. Of the total 27 CpG sites, 22 were methylated at varying ratios (Fig. 2). The average methylation ratio of the miR-193a gene in MG63.2 cells was approximately 20-fold higher than that in MG63 cells (58.93 vs. 3.38; Fig. 2c). Thus, increased methylation was negatively correlated with the expression of both miR-193a-3p and miR-193a-5p.

### MiR-193a-3p and miR-193a-5p suppress osteosarcoma cell migration and invasion in vitro

**Fig. 3:**
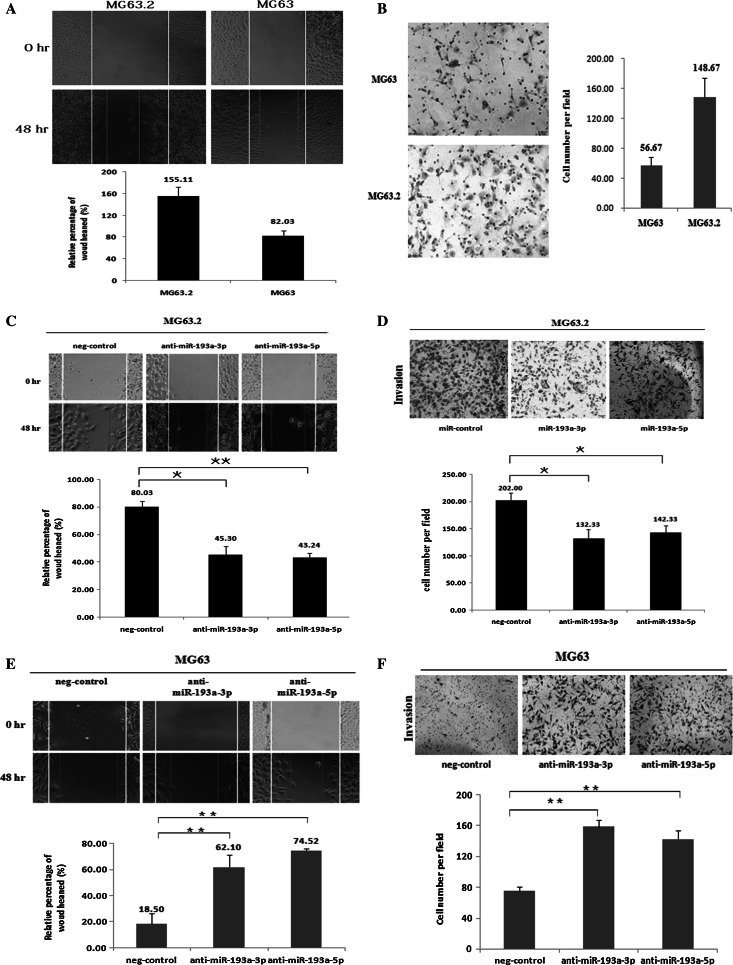
MiR-193a-3p and miR-193a-5p expression levels are reduced in highly metastatic cancer cells, consequently affecting cell migration and invasion.MG63.2 cells showed a higher capacity for migration and invasion than MG63 cells (**a, b**). Wound-healing assays that determine the migration and invasion of MG63 and MG63.2 cells were performed with transient expression of the miR-193a-3p mimic (3PM), miR-193a-5p (5PM) mimic, or negative control (NC) in MG63.2 cells as well as with the miR-193a-3p antagomiR (3PA), miR-193a-5p antagomiR (5PA) or NC (in MG63 cells) (**c**, **d**). Invasion assays for MG63.2 and MG63 cells were conducted after transduction with the same set of constructs (**e**, **f**). The data are representative of three independent experiments. *P < 0.05; **P < 0.01 by Student’s *t* test

The differential methylation status and expression of miR-193a-3p and miR-193a-5p between MG63 and MG63.2 cells indicate their potential roles in the metastasis of osteosarcoma. Therefore, we compared the migration and invasion capabilities of MG63.2 and MG63 cells using wound-healing and Matrigel invasion assays, respectively. As previously reported, MG63.2 cells showed a higher capacity for migration and invasion than MG63 cells (Fig. 3a, b), suggesting the active metastatic ability of MG63.2 cells. Loss- and gain-of-function studies of miR-193a-3p and miR-193a-5p were conducted by transiently transfecting miR-193a-3p and miR-193a-5p mimics or antagomiRs into MG63.2 and MG63 cells. As shown in Fig. 3c, d, both the migration and invasion of miR-193a-3p and miR-193a-5p mimic-transfected MG63.2 cells were significantly suppressed compared with those of control cells. In contrast, the migration and invasion of MG63 cells were increased by transfection of miR-193a-3p and miR-193a-5p antagomiRs (Fig. 3e, f).

### Rab27B and SRR are direct targets of miR-193a-3p and miR-193a-5p

**Fig. 4:**
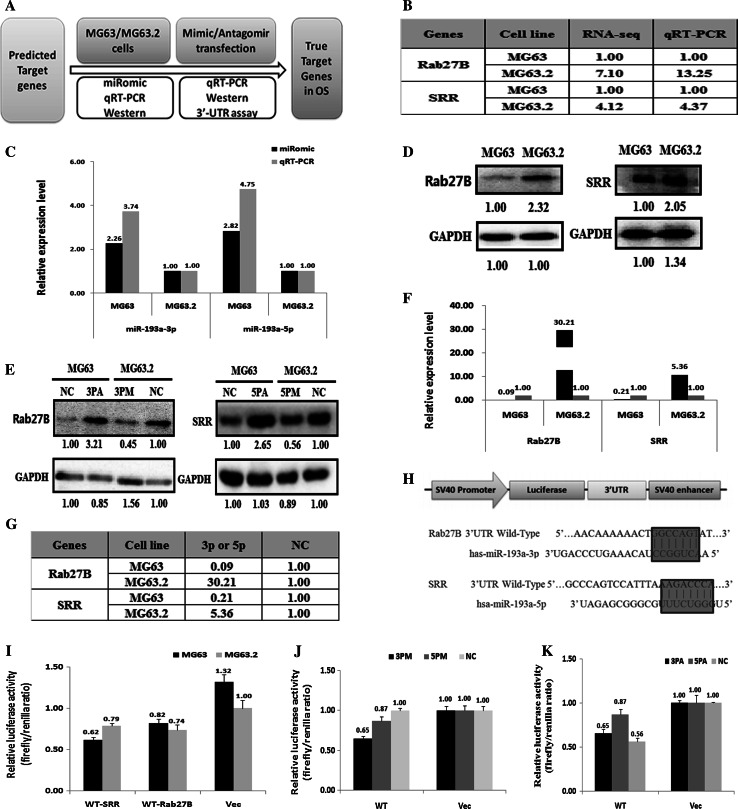
MiR-193a-3p and miR-193a-5p target gene expression is negatively correlated with miR-193a-3p and miR-193a-5p expression. **a** The experimental scheme. SRR and Rab27B gene expression in MG63 versus MG63.2 cells at the mRNA level based on RNA-Seq analysis (**b**) and qRT-PCR (**c**). SRR and Rab27B expression in MG63 versus MG63.2 cells at the protein level (**d**). SRR and Rab27B gene expression in miR-193a-3p or miR-193a-5p mimic (3PM/5PM)-transfected MG63.2 cells and miR-193a-3p or miR-193a-5p antagomiR (3PA/5PA)-transfected MG63 cells at the protein level as assessed by western blotting analysis (**e**) and at the mRNA level as assessed by qRT-PCR (**f**, **g**). **h** A schematic map of the pGL3-based luciferase reporter constructs where the UTR region (3′-UTR) of SRR or Rab27B gene was incorporated downstream of the luciferase gene. The relative luciferase activity (fold) of pGL3 with the Rab27B-UTR or SRR-UTR sequence relative to the control vector (VEC, with no UTR sequence) was determined in cells transfected with the miR-193a-3p mimic (3PM), antagomiR (3PA) or scramble negative control (NC) (**i**–**k**). Representative results from three independent experiments are shown. *P < 0.05; **P < 0.01 by Student’s *t* test

To further clarify the underlying molecular mechanisms of the suppressive effects of miR-193a-3p and miR-193a-5p on osteosarcoma cell metastasis, the predicted target genes of miR-193a-3p and miR-193a-5p were selected by using three commonly used miR-target-predicting methods as follows: miRDB, miRBase, and TargetScan (Fig. 4a). RNA-Seq data of MG63 and MG63.2 cells were then used to screen the possible candidates predicted to be expressed with the opposite trend to that of miR-193a-3p and miR-193a-5p between MG63 and MG63.2 cells (Fig. 4b, c). Subsequent qRT-PCR assays verified that the mRNA expression of Rab27B (target of miR-193a-3p) and SRR (target of miR-193a-5p), both from the candidate list, was significantly higher in MG63.2 cells than in MG63 cells (Fig. 4b, c). Western blot analysis also suggested that Rab27B and SRR protein levels were relatively higher in MG63.2 cells than that in MG63 cells (Fig. 4d). All of these results correlated well with the negative regulation of the target genes by miR-193a-3p and miR-193a-5p.

We next tested Rab27B and SRR expression with the addition of miR-193a-3p and miR-193a-5p mimics (termed 3PM and 5PM, respectively) in MG63.2 cells or miR-193a-3p and miR-193a-5p antagomiRs (termed 3PA and 5PA, respectively) in MG63 cells. Both mRNA and protein levels of Rab27B were suppressed by 3PM in MG63.2 cells and increased by 3PA in MG63 cells compared with those in control cells (Fig. 4e–g). Similarly, the mRNA and protein levels of SRR were suppressed by 5PM in MG63.2 cells and increased by 5PA in MG63 cells compared with those in control cells (Fig. 4e–g). These results suggested that Rab27B is the target of miR-193a-3p and that SRR is the target of miR-193a-5p.

To further determine if Rab27B and SRR are the direct targets of miR-193a-3p and miR-193a-5p, respectively, we searched for the miR-193a-3p- and miR-193a-5p-binding sites in the 3′-UTRs of Rab27B and SRR and generated pGL3-Rab27B UTR wild type (WT) and pGL3-SRR UTR WT constructs (Fig. 4h). Both constructs and the pGL3-control were transfected into MG63 and MG63.2 cells to determine if the differential expression of miR-193a-3p and miR-193a-5p was functional in the context of the two cell lines. Both UTR-containing constructs showed comparable luciferase activities in MG63.2 and MG63 cells (Fig. 4i). The transfection of 3PM and 5PM in MG63.2 cells significantly decreased the luciferase activity of the Rab27B-UTR and SRR-UTR constructs, respectively, compared with that in control cells (Fig. 4j). In contrast, the transfection of 3PA and 5PA in MG63 cells increased the luciferase activities of the Rab27B-UTR and SRR-UTR constructs, respectively, compared with the level in control cells (Fig. 4k). Thus, miR-193a-3p and miR-193a-5p decrease the expression of Rab27B and SRR, respectively, by directly targeting their 3′-UTRs.

### Rab27B and SRR are involved in the miR-193a-3p- and miR-193a-5p-induced suppression of osteosarcoma cell migration and invasion

**Fig. 5:**
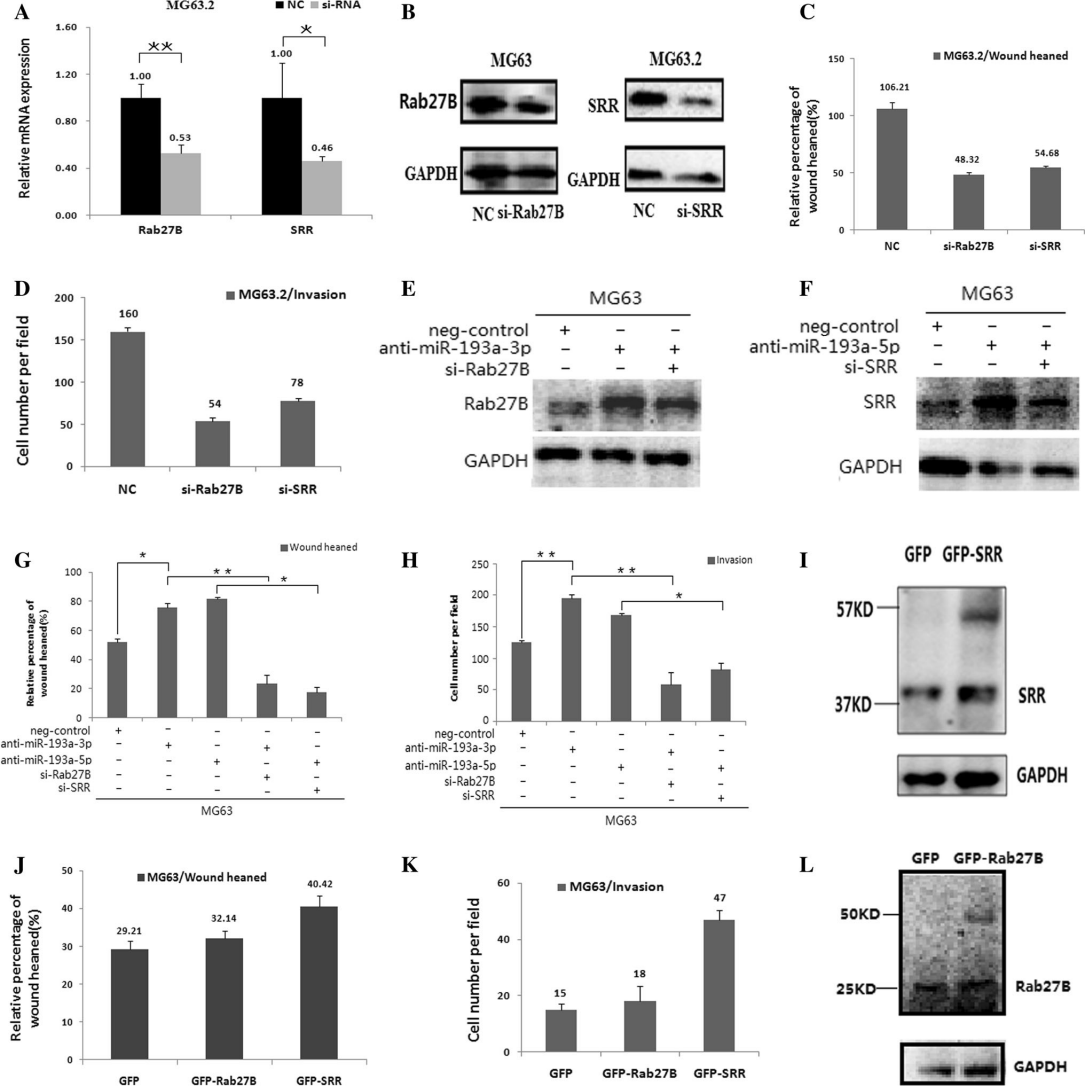
Rab27B and SRR are involved in the miR-193a-3p- and miR-193a-5p-induced suppression of osteosarcoma cell migration and invasion. Real-time PCR and western blot analyses of Rab27B and SRR expression in MG63.2 cells transfected with si-Rab27B, si-SRR or the negative control (NC). GAPDH was used as an internal control (**a**, **b**). Wound-healing and invasion assays were performed in MG63.2 cells after transfection with NC, si-Rab27B, si-SRR (**c**, **d**). Western blot analysis of Rab27B and SRR in MG63 cells stably expressing miR-193a-3p or miR-193a-5p antagomiR after transfection with si-Rab27B, si-SRR or NC. GAPDH was used as an internal control (**e**, **f**). Wound-healing and invasion assays using MG63 cells stably expressing miR-193a-3p or miR-193a-5p antagomiR after transfection with si-Rab27B, si-SRR or NC (**g**, **h**). The data are representative of three independent experiments. Western blot analysis of Rab27B and SRR in MG63 cells stably overexpressing GFP-tagged Rab27B and SRR proteins or GFP alone. GAPDH was used as an internal control (**i**, **j**). Wound-healing and invasion assays using MG63 cells stably overexpressing GFP-tagged Rab27B and SRR proteins or GFP (**k**, **l**). The data are representative of three independent experiments. *P < 0.05; **P < 0.01 by Student’s *t* test

To clarify the biological roles of Rab27B and SRR in osteosarcoma cells, we knocked down endogenous Rab27B and SRR expression using specific small interfering RNAs (siRNAs) in MG63.2 cells. As shown in Fig. 5a, b, Rab27B and SRR mRNA and protein levels were significantly reduced by the siRNAs. In addition, wound-healing and invasion assays showed that metastatic MG63.2 cell migration and invasion were also markedly inhibited by the transfection of both si-Rab27B and si-SRR (Fig. 5c, d).

We next determined if Rab27B and SRR dysregulation was involved in the miR-193a-3p- and miR-193a-5p-induced migration and invasion of osteosarcoma cells. The 3PA and si-Rab27B constructs were transfected separately or together into MG63 cells. As shown by western blot analysis in Fig. 5e, f, Rab27B expression was drastically increased by the transfection of 3PA in MG63 cells, whereas the co-transfection of both 3PA and si-Rab27B rescued Rab27B expression as well as the migration and invasion abilities of the cells (Fig. 5e, g, h). Similar results were also found with SRR in MG63 cells transfected with 5PA and/or si-SRR (Fig. 5f–h).

Furthermore, GFP-Rab27B and GFP-SRR proteins were over-expressed in MG63 cells. The ectopic expression of either Rab27B or SRR promoted MG63 cell migration and invasion (Fig. 5i–l).

Taken together, these results suggested that Rab27B and SRR are direct and functional targets of miR-193a-3p and miR-193a-5p, respectively, and that they are involved in the miRNA-induced suppression of osteosarcoma cell migration and invasion.

### MiR-193a-3p and miR-193a-5p regulate the TGFβ, Myc/Max and ATF2/ATF3/ATF4 signaling pathways involved in osteosarcoma metastasis

**Fig. 6:**
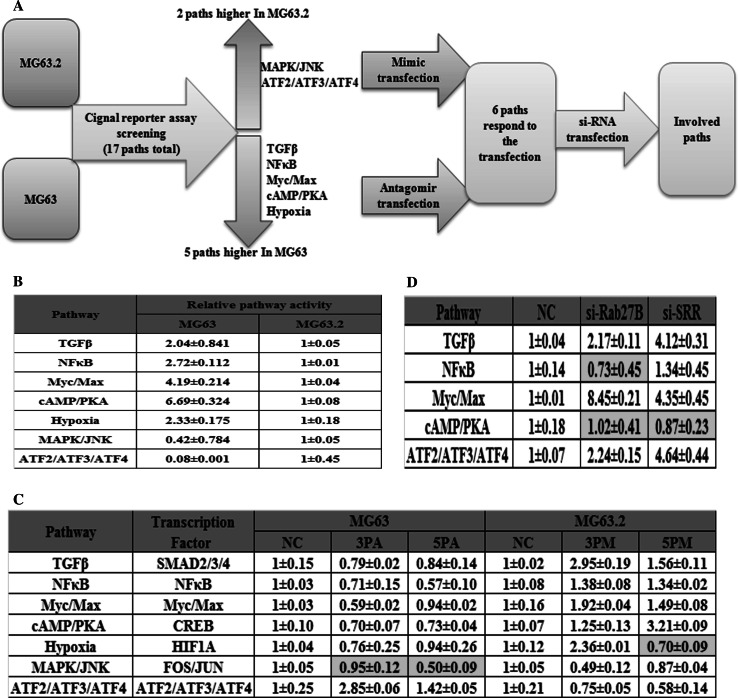
The signaling pathways regulated by miR-193a-3p, miR-193a-5p and their downstream genes. The experimental scheme (**a**). The relative activities (mean ± SD) of seven pathways that differed by more than two-fold between MG63 and MG63.2 cells (**b**). The relative pathway activities in the miR-193a-3p and miR-193a-5p mimic (3PM/5PM)-transfected versus NC-transfected MG63.2 cells as well as miR-193a-3p and miR-193a-5p antagomiR (3PA/5PA)-transfected versus NC-transfected MG63 cells (**c**). The relative pathway activities in the si-Rab27B- and si-SRR-transfected versus NC-transfected MG63.2 cells (**d**)

To gain further mechanistic insights into the roles of these miRNAs, we used the Cignal reporter finder assay (Fig. 6a) to compare the activities of 17 signaling pathways in MG63.2 versus MG63 cells. The MAPK/JNK and ATF2/ATF3/ATF4 pathway activities were 2-fold and 12-fold higher, respectively, in MG63.2 cells than inMG63 cells (Fig. 6b). In contrast, the TGFβ, NF-κB, Myc/Max, cAMP/PKA and hypoxia pathway activities were significantly lower in MG63.2 cells than in MG63 cells (Fig. 6b). These seven differentially regulated pathways might play a significant role in osteosarcoma metastasis. Therefore, we compared the activities of these pathways in mimic-transfected MG63.2 cells and antagomiR-transfected MG63 cells against the control cells. The TGFβ, NF-κB, Myc/Max, and cAMP/PKA pathways were down-regulated in the 3PA- or 5PA-transfected MG63 cells but up-regulated in the 3PM- or 5PM-transfected MG63.2 cells. The reverse effect was observed with the ATF2/ATF3/ATF4 pathway, which was activated in the 3PA- or 5PA-transfected MG63 cells and repressed in the 3PM- or 5PM-transfected MG63.2 cells (Fig. 6c). Furthermore, we compared the pathway activities inRab27B and SRR siRNA-transfected versus mock siRNA-transfected MG63.2 cells. Although no or marginal effects on the NF-κB and cAMP/PKA pathway activities were observed, the TGFβ, Myc/Max and ATF2/ATF3/ATF4 pathways were activated in either Rab27B or SRR siRNA-transfected MG63.2 cells (Fig. 6d). Notably, the Myc/Max pathway responded most drastically to Rab27B siRNA, whereas the ATF2/ATF3/ATF4 pathway was up-regulated to the greatest extent by SRR siRNA. Therefore, the role of Rab27B/miR-193a-3p is mainly accomplished via its effect on the Myc/Max pathway, whereas the role of SRR/miR-193a-5p is mainly accomplished via the ATF2/ATF3/ATF4 pathway.

**Fig. 7:**
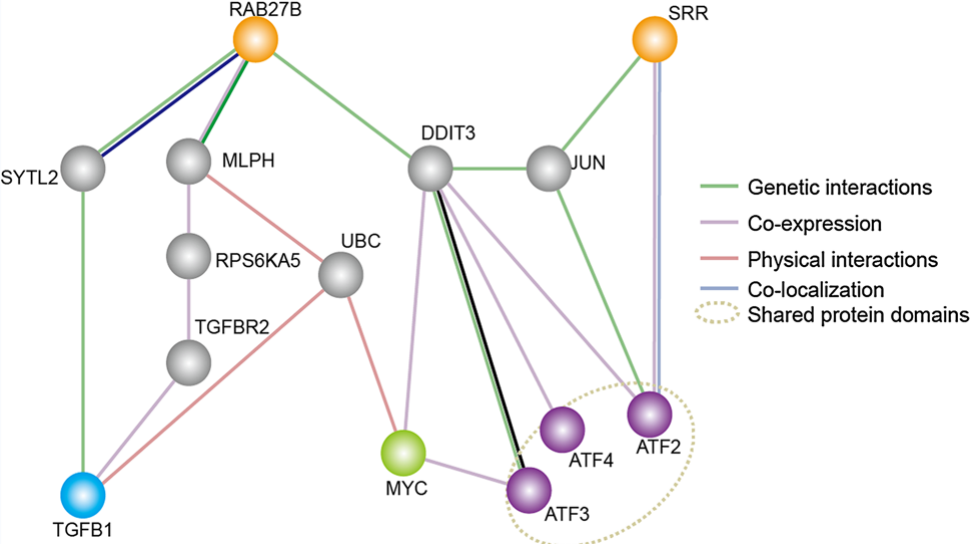
A simplified interaction map was analyzed between the Rab27B and SRR target genes and the TGFβ, Myc/Max and ATF2/ATF3/ATF4 pathways using GeneMANIA (http://genemania.org/). *Orange nodes* represent the target genes. *Gray nodes* represent the genes that were related to target genes according to the GeneMANIA method. *Blue*, *green* and *violet nodes* represent the master transcription factor genes for the TGFβ, Myc/Max and ATF2/ATF3/ATF4 pathways, respectively. The web-based interface searches yielded a large set of functional association data to return related genes based on available genomic and proteomic data. The association data include protein, DNA and genetic interactions as well as pathway data, gene expression data, protein expression data, phenotypic screens and shared protein domains. (Color figure online)

We then searched for interactions between the two target genes and the following master transcription factor genes of three signaling pathways (Fig. 7) using GeneMANIA (http://genemania.org/) [34]: TGFB1 for the TGFβ pathway, MYC for the Myc/Max pathway, and ATF2/ATF3/ATF4 for the ATF2/ATF3/ATF4 pathway. Both RAB27B and SRR genetically interacted with ATF2/ATF3/ATF4 via DDIT3 and JUN, respectively. In contrast, the interaction of RAB27B and SRR with MYC was directed by the co-expression of DDIT3 and MYC. The interaction of RAB27B and TGFB1 occurred via three genes as follows: SYTL2, MLPH and UBC. Despite the lack of direct interaction between SRR and TGFB1, SRR may affect the TGFβ pathway through agenetic interaction with RAB27B. All of these results explain why RAB27B and SRR, the target genes of miR-193a-3p and miR-193a-5p, respectively, regulate the three afore mentioned signaling pathways.

## Discussion

Aberrant miR-193a expression has been reported in many types of cancer, including colorectal cancer [33], NSCLC [24], epithelial ovarian cancer [35], myeloid leukemia [36] and Wilms’ tumors [37]. In addition, according to previous studies, miR-193a-3p is also involved in cancer drug resistance through the repression of different target genes [26, 38]. Here, we revealed for the first time that miR-193a-3p and miR-193a-5p are also involved in the suppression of osteosarcoma metastasis through two newly identified target genes, namely Rab27B and SRR.

MicroRNAs execute their biological function by repressing multiple genes at both the mRNA stability and translational levels. MiR-193a-3p and miR-193a-5p have been reported to target different genes that govern various types of cancer [27, 39]. In the present study, we showed that miR-193a-3p and miR-193a-5p were down-regulated in highly metastatic MG63.2 cells. The down-regulation of miR-193a-3p and miR-193a-5p correlated with the hyper-methylated state of their promoter and enhancer regions in MG63.2 cells (Figs. 1, 2). Previous studies have indicated that DNA methylation is the best characterized epigenetic mechanism, and it is regarded as a promising molecular indicator for the existence and/or the prognostic state of cancer [40, 41]. We next investigated target gene expression in highly metastatic MG63.2 cells compared with that in weakly metastatic MG63 cells. As expected, Rab27B and SRR mRNA and protein expression levels were increased in MG63.2 cells compared to MG63 cells (Fig. 4b–d). Moreover, increased Rab27B and SRR expression was correlated with decreased miR-193a-3p and miR-193a-5p expression (Fig. 4e–g). Hence, miR-193a-3p and miR-193a-5p negatively regulate osteosarcoma metastasis by targeting Rab27B and SRR, respectively.

Rab27B is a member of the RabGTPases, which constitute the largest family of small GTPases and play a vital role in endocytosis and exocytosis vesicle-trafficking control [42–45]. In addition, Rab27B proteins have been reported to be positively correlated with invasion and metastasis in colorectal cancer [46], hepatocellular carcinoma [47] and breast cancer [48–50], and these proteins mediate exosome secretion of tumor-related miRNAs and proteins such asproteases [51]. In agreement with these studies, our data suggested that Rab27B might facilitate the invasive/metastatic phenotypes of osteosarcoma and might thus represent a novel marker for clinical diagnosis and prognosis. SRR, the other identified gene, encodes a protein that catalyzes the synthesis of d-serine from l-serine. d-serine is a key co-agonist with glutamate at *N*-methyl-d-aspartate (NMDA) receptors. Although no direct evidence has indicated a relationship between SRR and metastasis, NMDA receptors are associated with cell migration [52] and metastasis in oral squamous cell carcinoma [53]. Our work suggested for the first time that miR-193a-3p-regulated Rab27B and miR-193a-5p-regulated SRR contribute to the invasion and metastasis of osteosarcoma. However, the detailed mechanism awaits further investigation.

MiR-193a-3p and miR-193a-5p, two mature products from an identical precursor RNA, have previously been reported to suppress the metastasis of human NSCLC by down-regulating the ERBB4/PIK3R3/mTOR/S6K2 signaling pathway [28]. Additionally, miR-193a-5p depletion has been characterized as a marker of metastasis and poor prognosis in colorectal cancer [54]. Our present study showed for the first time that miR-193a-3p and miR-193a-5p can suppress human osteosarcoma cell metastasis by suppressing two novel targets, Rab27B and SRR, respectively. Moreover, knocking down SRR inhibited the TGF-β, NF-κB, Myc/Max and ATF2/3/4 pathway activities, whereas knocking down Rab27B repressed the same three pathways, except for the NF-κB pathway, partially mimicking the effects of miR-193a-3p- and miR-193a-5p-mimic transfection. The TGF-β and Myc/Max pathways are well known for their positive impact on cancer metastasis [55, 56]. ATF4 hasbeen reported to promote esophageal squamous cell carcinoma invasion and metastasis [57], whereas the effect of ATF3 on metastasis in different types of cancer remains controversial [58–61]. By inhibiting these pathways, Rab27B and SRR could be effective and functional downstream targets of miR-193a-3p and miR-193a-5p in the mechanism of cancer metastasis. However, more details of this process and whether there is a synergistic effect between miR-193a-3p and miR-193a-5p as well as their targets requires further investigation.

In this study, we demonstrated that miR-193a-3p and miR-193a-5p suppress the metastasis of human osteosarcoma cells by repressing Rab27B and SRR expression, through suppressing the TGFβ, Myc/Max and ATF2/ATF3/ATF4 signaling pathways. This study also provided a new set of genes that may be useful biomarkers for the diagnosis of osteosarcoma.

## Abbreviations

OS: Osteosarcoma
MiR: MicroRNA
UTR: Untranslated region
Rab27B: Member RAS oncogene family
SRR: Serine racemase
3PM/5PM: MiR-193a-3p and miR-193a-5p mimic
3PA/5PA: MiR-193a-3p and miR-193a-5p
BSP: Bisulfite sequencing PCR
